# Survival of *Salmonella* and *Listeria monocytogenes* on Food Contact Surfaces in Produce Packinghouses

**DOI:** 10.3390/foods14183247

**Published:** 2025-09-18

**Authors:** Cyril A. Etaka, Eugenia M. Silva, Alexis M. Hamilton, Claire M. Murphy, Laura K. Strawn

**Affiliations:** 1 Department of Food Science and Technology, Virginia Tech, Blacksburg, VA 24061, USA; cyrilnsom@vt.edu (C.A.E.);; 2 School of Food Science, Washington State University Irrigated Agricultural Research and Extension Center, Prosser, WA 99350, USA; claire.murphy@wsu.edu

**Keywords:** *Salmonella*, *Listeria monocytogenes*, food contact surfaces, biphasic model, packinghouse, pathogen persistence, cross-contamination, sanitation, clean breaks

## Abstract

Short-season (90 d) produce packing operations may run double shifts with no clean breaks in between. This practice can result in produce contamination from food contact surfaces that are not cleaned and sanitized. Our study examined the survival of *Salmonella* and *Listeria monocytogenes* on polycarbonate, polypropylene, polyvinyl chloride (PVC), rubber, and stainless steel surfaces that contact produce in operations that have a short packing season. Coupons were spot-inoculated with five-strain cocktails of rifampicin-resistant *Salmonella* or *L. monocytogenes* (~7 log CFU/coupon), stored at 22 °C and 45–55% relative humidity, and enumerated at 0, 0.06, 0.25, 1, 2, 3, 7, 10, 14, 21, 30, 60, and 90 d. Significant differences were evaluated (*p* ≤ 0.05), and survival was modeled using linear and biphasic models. *Salmonella* reductions varied significantly by surface type, with rubber showing the greatest survival, followed by stainless steel at 90 d. In contrast, *Salmonella* concentrations on polycarbonate, polypropylene, and PVC were below the limit of detection at 90 d. *L. monocytogenes* reductions were not significantly different across materials at 90 d. Biphasic models better fit the inactivation of both pathogens. These findings highlight the importance of clean breaks and focusing interventions where pathogens demonstrate greater persistence in short-season packinghouses.

## 1. Introduction

Stainless steel, various plastics [e.g., polycarbonate, polypropylene, and polyvinyl chloride (PVC)], and rubber are widely used as food contact surfaces in fresh produce operations [1]. Stainless steel is often preferred for its durability, corrosion resistance, and smooth finish, which facilitates cleaning and sanitization [2]. Plastic and rubber components are commonly found in equipment such as conveyor belts, gravity rollers, gaskets, gloves, and brush filaments [3].

According to prior studies, pathogens of concern for the produce industry can survive on stainless steel, plastic, and rubber materials used to make produce food contact surfaces, with different factors such as surface type, surface condition (clean or soiled), porosity, relative humidity, temperature, and others impacting bacterial survival [4,5,6,7,8,9]. For example, *Salmonella* was found to persist on conveyor belt, PVC, and stainless-steel materials for approximately 3 to 28 d based on holding temperature and RH [6]. Similarly, *Salmonella* was recovered from stainless steel surfaces after a 4-d holding time, although persistence decreased with decreasing initial inoculum [7]. In a different study, *L. monocytogenes* survived on clean conveyor belt materials (PVC, polyurethane, nitrile rubber) for approximately 4 to 10 d but persisted for 14 d on clean foam pads and nylon brush [8]. When surfaces were soiled with cantaloupe extract, the population of *L. monocytogenes* remained unchanged on all soiled surfaces for 14 d [8].

Bacteria that survive on food contact surfaces can be transferred to fresh produce during routine handling and processing, potentially leading to outbreaks that are traceable to these contaminated surfaces [8,10,11,12]. For example, a traceback investigation of the 2011 multistate listeriosis outbreak linked to whole cantaloupes identified *L. monocytogenes* on packing equipment surfaces, which directly contaminated cantaloupe rinds and led to 147 illnesses and 33 deaths [13]. In the case of the caramel apple listeriosis outbreak (34 hospitalized cases and 7 deaths), polishing and drying brushes and the conveyor belt were implicated [14]. Similarly, traceback investigations during the *Salmonella*-associated onion outbreak identified inadequate cleaning, maintenance, and inspection of food contact surfaces as likely contributors to the spread of *Salmonella* in onion harvesting and packing operations [15].

Surfaces that facilitate long-term pathogen persistence can act as ongoing sources of contamination, especially when cleaning is limited by operational constraints (e.g., compressed schedules during short harvest seasons, resource availability). For example, produce with short packing seasons can run double shifts (e.g., 16 to 20-h days), leaving a limited time for daily cleaning and sanitization. As a result, full equipment disassembly and cleaning are performed, when possible, at the beginning and/or end of the packing season, which may allow microbial harborage sites to persist throughout production.

Despite recognition of these risks, limited data exist on survival and persistence of bacterial pathogens, such as *Salmonella* and *L. monocytogenes*, across diverse food contact surface materials under produce packinghouse conditions, particularly in operations with short produce packing seasons (over 90 d). These data, along with survival modelling, are needed to inform hazard identification, risk assessment, and the design of surface-specific mitigation strategies in such operations. Therefore, the objective of this study was to investigate and model the survival of *Salmonella* and *L. monocytogenes* on five commonly used food contact surfaces (polycarbonate, polypropylene, PVC, rubber, and stainless steel) commonly observed in operations with a short produce packing season (over a 90 d duration).

## 2. Materials and Methods

### 2.1. Bacterial Strains and Inoculum Preparation

The *Salmonella enterica* cocktail consisted of five serovars previously linked to produce-related outbreaks: Agona ATCC BAA-707 (2011 alfalfa outbreak; [16]), Enteritidis 2020AM-1539 (2020 peach outbreak; [17]), Montevideo ATCC BAA-710 (1993 tomato outbreak; [18]), Newport 2020AM-0919, (2020 onion outbreak; [19]), and St. Paul (2008 pepper outbreak; [20]). Additionally, the five strains used to prepare the *L. monocytogenes* cocktail included: LM LS320 (2016 frozen broccoli recall; [21]), LM 390-2 (2011 cantaloupe outbreak; [13]), LM 390-6 (2011 cantaloupe outbreak; [13]), Scott A (1983 pasteurized milk outbreak; [22]), and LM 573-035 (2014 caramel apple outbreak; [14]). All strains were adapted to grow in the presence of 80 μg/mL rifampicin (Fisher Scientific, Fair Lawn, NJ, USA) to obtain antibiotic-resistant strains. These antibiotic-resistant strains were used to minimize interference with background bacteria and facilitate the recovery of all antibiotic-resistant strains on rifampicin-amended media. The strains were held in 15% glycerol at −80 °C prior to inoculum preparation.

Inoculum preparation for both pathogens was adapted from Danyluk et al. [23]. The frozen stock culture of each bacterial strain was streaked on Tryptic Soy Agar containing 80 μg/mL of rifampicin (TSAR; Difco, Becton Dickinson Co., Sparks, MD, USA) and incubated at 35 °C for 24 h. An isolated colony from TSAR plates was transferred to 10 mL Tryptic Soy Broth containing 80 μg/mL rifampicin (TSBR; Difco, Becton Dickinson Co., Sparks, MD, USA) and incubated at 35 °C for 24 h. After incubation, a 10 μL loopful of overnight culture was transferred to 10 mL of fresh TSBR. The second overnight culture of each strain (100 μL) was spread across separate TSAR plates with a sterile L-shaped spreader and incubated at 35 °C for 24 h. The resulting bacterial lawn of each strain was harvested by adding 5 mL 0.1% peptone water, scraping the lawn with a sterile spreader, and pipetting the slurry of each strain into separate sterile Falcon tubes (Fisher Scientific, Fair Lawn, NJ, USA). The culture slurry of each strain was combined in equal amounts (2 mL per strain) to obtain the cocktails. The slurry concentration was adjusted by diluting two-fold in 0.1% peptone water to reach an approximate starting inoculum concentration of 8 log CFU/mL. Final concentrations of the inoculum were verified by enumeration on TSAR.

### 2.2. Coupon Preparation and Inoculation

For each pathogen, *Salmonella* and *L. monocytogenes,* the present study included duplicate trials with five samples per material type analyzed at each of the 13 time points, resulting in a total of 260 samples. Before trials, food contact surfaces (Polycarbonate, Polypropylene, PVC, Rubber, and Stainless Steel 316; [Biosurface Technologies, Bozeman, Montana]) were cut into 25 cm^2^ coupons and surface-sterilized by dipping in 70% ethanol for 5 min, patting dry with paper towels, placing in weigh boats, and exposing to UV light for 5 min in a biosafety cabinet (United Scientific Supplies Inc., Libertyville, IL, USA). Coupons were spot-inoculated with a rifampicin-resistant five-strain cocktail of either *Salmonella* or *L. monocytogenes* by distributing 100 μL of inoculum in 15 to 25 droplets. These surfaces were left in a biosafety cabinet to dry for 1.5 h. Once dry, coupons were held in a controlled environment (Percival Scientific, Inc., Perry, IA, USA) at 22 °C and 45–55% RH for up to 90 d. These holding conditions were selected to simulate the average temperature and RH in a packinghouse facility located in the Mid-Atlantic produce region of the United States. Holding parameters were recorded using data loggers (Spectrum Technologies Inc., Thayer Court, Aurora, IL, USA) to check for consistency in holding temperature (<2 °C fluctuation) and RH (approximately 10% fluctuation). Enumeration of bacteria on coupons was performed at 13 time points (0, 1.5, 6 h, and 1, 2, 3, 7, 10, 14, 21, 30, 60, and 90 d).

### 2.3. Coupon Enumeration

At each time point, coupons were transferred to Whirl-Pak bags (Nasco, Fort Atkinson, WI, USA) containing 20 mL of 0.1% peptone water + 1% Tween80 (Fisher Scientific, Fair Lawn, NJ, USA). The samples were hand-massaged and shaken for 1 min. Serial dilutions of the rinsate were performed with 0.1% peptone water dilution blanks (9 mL) and appropriate dilutions were plated onto non-selective media (TSAR) for both pathogens and selective media for *Salmonella* [Rifampicin-amended Xylose Lysine Deoxycholate (XLDR); Fisher Scientific, Fair Lawn, NJ, USA] or *L*. *monocytogenes* [Rifampicin-amended Modified Oxford Agar (MOXR); Fisher Scientific, Fair Lawn, NJ, USA]. All plates were held at 35 °C for 24 h (*Salmonella*) or 48 h (*L. monocytogenes*), and counts were expressed in log CFU/coupon. When counts fell below the limit of detection (<1.30 log CFU), enrichments were performed following a modified version of the Food and Drug Administration’s Bacteriological Analytical Manual (FDA BAM; [24]). Briefly, 1 mL of coupon rinsate was transferred to 9 mL of Buffered Peptone Water (BPW; Fisher Scientific, Fair Lawn, NJ, USA) and incubated at 35 °C for 24 h. From the pre-enrichment, 100 μL was transferred to 10 mL of Rappaport Vassiliadis Broth (RV; Becton Dickinson CO., Sparks, MD, USA) and incubated at 35 °C for 24 h for *Salmonella* enrichment. For *L. monocytogenes*, pre-enrichment was performed in BPW as previously described, and 1 mL of overnight pre-enrichment was transferred into 9 mL of Buffered *Listeria* Enrichment Broth (BLEB; Difco, Becton Dickinson CO., Sparks, MD, USA) and incubated at 35 °C for 24 h. A 10-μL loopful of the RV broth was then streaked onto XLDR agar plates to confirm *Salmonella* presence or absence. Overnight enrichments in BLEB were streaked on MOXR to determine whether *L. monocytogenes* was present or absent.

### 2.4. Statistical Analysis

All analyses were performed in R version 4.0.2 (R Foundation for Statistical Computing). The mean and standard deviation in log CFU/coupon for the survival of *Salmonella* and *L. monocytogenes* were determined for every material type at each time point. A one-way ANOVA was used to assess overall differences, and significant differences over time and between material types at specific timepoints were evaluated using Tukey’s Honest Significance Difference test (*p* ≤ 0.05).

To characterize the die-off pattern of *Salmonella* and *L. monocytogenes* overall and on each material type, Log-linear and Biphasic die-off models were built.

i. Log-linear model [25]
  $$N_{t}=N_{0}\times {exp(-k}_{max}\times t)$$
  where *N_t_* is the population (Log CFU/coupon) at time *t* (days), *N*_0_ is the initial inoculum at 0 h, and *k_max_* is the rate of decline (in days).

ii. Biphasic die-off model [26]
  $${Log}_{10}\left(N_{t}\right)={Log}_{10}\left(N_{0}\right)+{Log}_{10}\left[f\times {exp(-k}_{max1}\times t\right)+\left(1-f\right)\;\times {exp(-k}_{max2}\times t)]$$
  where *N_t_*, *t*, and *N*_0_ are defined above, the ƒ parameter is the fraction of the initial population that dies off before the breakpoint, *k_max_*_1_ is the rate of decline (days) before the breakpoint, and *k_max_*_2_ is the rate of decline (days) after the breakpoint.

Goodness of fit between models was evaluated using the Akaike Information Criterion (AIC). Lower AIC values indicate a better-fitting model. The change in AIC (delta AIC) was calculated as the difference between the AIC of each model and the model with the lowest AIC.

## 3. Results and Discussion

For both *Salmonella* and *L. monocytogenes*, significant differences were observed between counts on selective and non-selective plates. This difference between media types was attributed to the inclusion of reagents in the selective media types (XLDR and MOXR) that select healthy uninjured cells [27,28,29]. Significant differences between selective and non-selective media types for the recovery of bacterial cells have previously been observed [5,28,30,31]. Therefore, data were analyzed separately, with the non-selective plate results presented below and selective results included in the Supplemental Materials Tables S1 and S2.

Following inoculation, the mean starting concentration (0 d) of *Salmonella* on coupons was approximately 6.9 log CFU/coupon (Table S3). After 1.5 h of drying (0.06 d), *Salmonella* concentration on polycarbonate reduced significantly (0.88 ± 0.09 log CFU/coupon reduction) compared to the other studied surfaces to a concentration of 5.94 ± 0.13 log CFU/coupon (*p* ≤ 0.05; Table 1 and Table S3). However, all surface types showed a significant reduction from 0 d to 0.06 d. After 1 d, *Salmonella* reductions on rubber coupons were significantly lower (0.97 ± 0.21 log CFU/coupon reduction to a concentration of 6.00 ± 0.21 log CFU/coupon) compared to all other surfaces (*p* ≤ 0.05). At 7 d, PVC and rubber had the highest and lowest reductions in *Salmonella*, at 2.47 ± 0.52 and 1.87 ± 0.54 log CFU/coupon, respectively (Table 1). The concentrations of PVC and rubber at this time-point (7 d) were 4.48 ± 0.52 and 5.10 ± 0.54 log CFU/coupon, respectively (Table S3). At 30 d, rubber with a concentration of 4.60 ± 0.19 log CFU/coupon still exhibited the lowest reduction, at 2.37 ± 0.19 log CFU/coupon, which was significantly different from the other surfaces (*p* ≤ 0.05; Table 1 and Table S3). At 90 d, *Salmonella* counts fell below the LOD (<1.30 log CFU/coupon) on all polycarbonate, polypropylene, and PVC coupons with reductions of approximately 5.6 log CFU/coupon, although detection of *Salmonella* by enrichment was still possible (Table 1). *Salmonella* remained quantifiable on stainless steel (1.50 ± 0.64 log CFU/coupon) and rubber (2.59 ± 1.11 log CFU/coupon), with reductions of 5.37 ± 0.65 and 4.39 ± 1.13 log CFU/coupon, respectively (Table 1 and Table S3). Overall survival of *Salmonella* on rubber at 90 d was significantly greater than on each of the other four surfaces (*p* ≤ 0.05; Table 1).

As for *L. monocytogenes*, initial concentrations following inoculation on coupon surfaces (day 0) were approximately 6.6 log CFU/coupon (Table S4). After 1.5 h of drying (0.06 d), all surfaces exhibited a significant reduction in *L. monocytogenes*, with the reduction on stainless steel being significantly greater than on the other four surfaces (1.79 ± 0.43 log CFU/coupon reduction to a concentration of 5.12 ± 0.43 log CFU/coupon; Table 2 and Table S4). By 7 d, the reductions varied across surfaces, with PVC at a concentration of 2.89 ± 0.33 log CFU/coupon, showing the greatest reduction (3.70 ± 0.33 log CFU/coupon) compared to all others tested, and polycarbonate at a concentration of 4.24 ± 0.10 log CFU/coupon, showing the lowest reduction (2.29 ± 0.10 log CFU/coupon; Table 2 and Table S4). Polycarbonate maintained the lowest reduction throughout the remainder of the study (Table 2). Unlike *Salmonella*, by 90 d, no significant differences in *L. monocytogenes* reductions were observed between any of the surfaces, with overall reductions averaging approximately 5.3 log CFU/coupon (Table 2).

The differential impact of surface (i.e., material type) on pathogen survival may be due to differences in microbial physiology and stress tolerance mechanisms. *L. monocytogenes* is generally more resilient to acid conditions, low-water-activity environments, desiccation, low temperatures, and others compared to *Salmonella* due to stress tolerance mechanisms that are under the control of an alternative sigma factor [32]. Additionally, *L. monocytogenes* exhibits different colonization behavior from other Gram-negative bacteria, like *E. coli*, on surfaces with intermediate roughness (hydrophobic) [33]. Even though our study objective did not cover surface characteristics, our findings suggest that they play a role in bacterial colonization and survival. Surfaces such as polycarbonate, polypropylene, and PVC are typically smoother, less porous, and hydrophobic, thus reducing microbial adhesion and moisture retention [34]. Rubber, by contrast, is more porous and rougher due to its fibrous, elastic matrix, which can trap moisture, organic matter, and bacterial cells in protected niches [33,35]. Stainless steel, although nonporous, can accumulate micro-scratches, which may shelter pathogens from environmental stressors [33]. Regardless of pathogen and surface characteristics, no growth was observed on all surfaces studied. This aligns with the Food Safety Modernization Act Produce Safety Rule, which requires that all food contact surfaces be constructed so they do not promote bacterial growth [36]. However, the bacterial microorganisms persisted over 90 d, with recovery by enrichment observed in some cases. These findings suggest that current practices in short-season produce packinghouses—where operational constraints may limit adequate cleaning and sanitization (e.g., between different lots of produce)—can pose significant risks for food contamination. This underscores the need for routine cleaning and sanitizing of all food contact surfaces within packinghouses to reduce the survival and spread of pathogens.

Based on the log-linear models comparing daily die-off (i.e., inactivation) rates between surfaces, the linear rate of decline in *Salmonella* on rubber was significantly slower than the other four tested surfaces in the present study (Table 3). No additional significant differences in daily linear die-off rates were observed between surfaces. Overall, the *k_max_* (rate of decline) for *Salmonella* from the linear models ranged from 0.038 log CFU/day (rubber) to 0.050 log CFU/day (polycarbonate and polypropylene; Table 4). Across the surfaces combined, *Salmonella* populations declined at an average rate of 0.047 log CFU/day (Table 4). When *Salmonella* was modeled using a biphasic approach, all surfaces exhibited an initial rapid die-off, with population reductions ranging from 1.259 log CFU/coupon on polycarbonate to 1.582 log CFU/coupon on PVC, followed by a slower decline (Table 4). This pattern was consistent across surfaces, as *k_max_*_1_ values were always greater than *k_max_*_2_ values (Table 4). When a biphasic model was run for all surfaces combined, the *Salmonella* population reduced by 1.379 log CFU/coupon at a rate of 0.828 log CFU/day, followed by a rate of decline of 0.037 log CFU/day for the remaining 5.067 log CFU/day. Based on the AIC and dAIC, the biphasic model better explained the shape of the *Salmonella* die-off pattern on all five surfaces combined and on each individual surface (Table 5).

No significant difference in the linear decline rates of *L. monocytogenes* was observed among the five surfaces examined in the present study (Table 3). Overall, the *k_max_* (rate of decline) for *L. monocytogenes* across the surfaces combined was 0.047 log CFU/day (Table 4). When *L. monocytogenes* was modeled using a biphasic approach, all surfaces exhibited an initial rapid die-off followed by a slower decline. The *k_max_*_1_ rates by material type ranged from 0.641 log CFU/day (on stainless steel) to 0.980 log CFU/day (on PVC), followed by *k_max_*_2_ values of 0.013 log CFU/day (on PVC) to 0.033 log CFU/day (on polycarbonate; Table 4). For *L. monocytogenes* on all surfaces combined, 2.273 log CFU/coupon of bacterial cells declined at a rate of 0.748 log CFU/day, with a subsequent decline of 0.023 log CFU/day for the remaining 2.986 log CFU/coupon of bacterial cells. Based on the AIC and dAIC, the biphasic model better explained the shape of the *L. monocytogenes* die-off pattern on all surfaces combined and on each surface (Table 5).

The die-off patterns of *Salmonella* and *L. monocytogenes* differed in both the log-linear and biphasic models (Table 4). In the log-linear model, *Salmonella* declined at rates ranging from 0.038 (rubber) to 0.050 (polycarbonate, polypropylene) log CFU/day, with rubber demonstrating a significantly slower decline, while *L. monocytogenes* showed a relatively uniform die-off across surfaces (Table 4). The biphasic model revealed a more variable rapid initial decline for *Salmonella*, with *k_max_*_1_ values highest on PVC (1.585 log CFU/coupon) and lowest on rubber (0.608 log CFU/coupon), whereas *L. monocytogenes* had its highest initial die-off rate on PVC (0.980 log CFU/coupon) and the lowest on polypropylene (0.660 log CFU/coupon; Table 5). *L. monocytogenes* exhibited lower *k_max_*_2_ values than *Salmonella*, particularly on PVC (0.013 log CFU/coupon; Table 4). However, differences in estimated starting populations (intercept) between the two pathogens may have influenced the observed die-off patterns, as *Salmonella* intercepts were higher than those of *L. monocytogenes* (Table 4).

A biphasic model pattern described bacterial die-off as non-linear, with two distinct phases: a first phase with a rapid initial die-off rate, followed by a tailing phase (or second phase) with a slower die-off rate [26,37]. Several studies have also demonstrated that bacterial die-off on surfaces follows a non-linear pattern [4,5,38,39,40,41,42]. For example, the die-off of *Salmonella* and *L. monocytogenes* on both clean and fouled stainless steel surfaces was best described by biphasic and Weibull models as compared to the linear model [4]. Similarly, the decline in *E. aerogenes*, a non-pathogenic surrogate for *Salmonella*, on stainless steel, PVC, and ceramic tiles held at 50% RH at 7 or 21 °C was best fit by biphasic and Weibull models [38]. Although these other studies evaluated bacterial survival for less than 90 d, they support the findings reported here, which observed *Salmonella* and *L. monocytogenes* die-off on the five tested surfaces followed a non-linear pattern. The present study, in combination with previous work, shows that linear assumptions may inaccurately predict bacterial persistence on surfaces. Therefore, scientific studies should consider non-linear approaches when investigating bacterial persistence and decay (i.e., inactivation, die-off) kinetics.

This study demonstrated that persistence of *Salmonella* and *L. monocytogenes* on surfaces for at least 90 d was influenced by both surface material type and pathogen, with differences in reduction patterns and rates of decline. *Salmonella* exhibited greater surface-dependent variability in survival than *L. monocytogenes*, particularly on rubber, where reductions were consistently lower across time-points. Allen et al. [6] also observed material-dependent differences in *Salmonella* persistence at 30 °C and 80% RH on stainless steel, conveyor belts, PVC, sponge rollers, and unfinished oak surfaces, respectively. The greater *Salmonella* survival on rubber may be due to its porous, fibrous structure, as discussed, as well as the colonization behavior of *Salmonella*. These findings are consistent with prior observations of *Salmonella* persistence on rubber gloves [4]. In this study, *Salmonella* exhibited slower die-off rates (*k_max_*_1_ and *k_max_*_2_) on clean rubber gloves inoculated with a wet inoculum than stainless steel surfaces under the same conditions, highlighting persistence for a longer duration [4]. Rubber may be a higher-risk surface for *Salmonella* in produce operations. Additionally, the slower decline rates for *Salmonella* on rubber may indicate greater harborage potential, suggesting targeted sanitation or equipment redesign. Operations may also perform a hazard analysis to assist in determining whether surface-specific interventions (e.g., cleaning regimens) would be appropriate.

Previous research has shown that factors such as surface condition (e.g., clean vs. soiled), inoculation method (e.g., wet vs. dry), and storage conditions (e.g., temperature and RH) may have a greater impact on pathogen survival than surface type alone [4,38,43,44]. The current study was conducted under controlled conditions using clean surfaces and a single temperature and RH level. In the packinghouse environment, food contact surfaces are often exposed to organic debris, water, and soil, all of which can negatively alter pathogen survival dynamics. As such, future studies should evaluate long-term persistence under variable conditions to better understand the compounding effects of surface material, condition, and environment.

## 4. Conclusions

The survival of both pathogens for at least 90 d highlights the potential cross-contamination risk in packinghouse environments if surfaces are not cleaned and sanitized during the packing season (for instance, between shifts). Our findings suggest that sanitation clean breaks could be scheduled more strategically based on surface-specific survival profiles. For instance, surfaces like rubber that support longer survival of *Salmonella* may need more frequent or targeted cleaning during long packing runs. Sanitation clean breaks are important to limit cross-contamination and reduce the scope of potential recalls. Sanitation practices (e.g., frequency, chemicals) should be evaluated, as well as pathogen survival under more variable environmental conditions (e.g., temperature fluctuations, extremes). These data also support the incorporation of non-linear modeling approaches to more accurately reflect pathogen decay dynamics (i.e., inactivation, die-off) on surfaces over extended storage durations. Ultimately, incorporating surface-specific survival data into packinghouse sanitation programs can reduce the risk of cross-contamination events and inform science-based cleaning schedules.

## Figures and Tables

**Table 1:** *Salmonella* concentration reductions (mean ± standard deviation in log CFU/coupon) on food contact surfaces under conditions of 45–55% RH, 22 °C enumerated on non-selective media (10 samples in each cell).

Days	Polycarbonate	Polypropylene	PVC	Rubber	Stainless Steel
0	0.00 ± 0.00 aA ^a^	0.00 ± 0.00 aA	0.00 ± 0.00 aA	0.00 ± 0.00 aA	0.00 ± 0.00 aA
0.06	0.88 ± 0.09 cB	0.67 ± 0.28 abB	0.76 ± 0.15 bB	0.53 ± 0.34 aB	0.45 ± 0.24 abB
0.25	0.98 ± 0.22 bBC	1.05 ± 0.20 bBC	1.03 ± 0.18 bB	0.69 ± 0.16 aB	1.02 ± 0.19 bC
1	1.24 ± 0.38 bcBCD	1.42 ± 0.26 bcCD	1.62 ± 0.38 cC	0.97 ± 0.21 aBC	1.13 ± 0.35 bCD
2	1.47 ± 0.44 bCD	1.77 ± 0.22 bDE	1.76 ± 0.45 bC	1.18 ± 0.27 aC	1.46 ± 0.23 bDE
3	1.70 ± 0.55 abDE	2.01 ± 0.22 bE	2.06 ± 0.39 bCD	1.69 ± 0.31 aD	1.74 ± 0.19 abEF
7	2.01 ± 0.54 abEF	2.08 ± 0.57 abE	2.47 ± 0.52 bDE	1.87 ± 0.54 aDE	1.98 ± 0.30 abFG
10	2.40 ± 0.57 bFG	2.25 ± 0.39 abEF	2.45 ± 0.58 bDE	2.01 ± 0.52 aDEF	2.39 ± 0.41 bGH
14	2.56 ± 0.37 bGH	2.73 ± 0.47 bFG	2.70 ± 0.49 bE	2.13 ± 0.39 aDEF	2.51 ± 0.51 bHI
21	2.56 ± 0.51 bG	2.78 ± 0.47 bG	2.82 ± 0.57 bEF	2.30 ± 0.29 aEF	2.56 ± 0.41 abHI
30	3.08 ± 0.57 bH	3.02 ± 0.46 bG	3.33 ± 0.60 bFG	2.37 ± 0.19 aF	2.86 ± 0.25 bI
60	4.17 ± 1.06 bI (1/3) ^b^	4.22 ± 1.14 bH (1/4)	3.84 ± 1.01 abG (4/7)	3.09 ± 0.42 aG	3.67 ± 0.79 abJ
90	5.56 ± 0.00 bJ (1/10)	5.66 ± 0.00 bI (3/10)	5.69 ± 0.00 bH (2/10)	4.39 ± 1.13 aH (4/5)	5.37 ± 0.65 bK (3/8)

^a^ Lowercase letters indicate significant differences (*p* ≤ 0.05) at a single time point (rows). Capital letters indicate a significant difference (*p* ≤ 0.05) within material type over time (columns). ^b^ Parentheses express the number of coupons with a positive enrichment result over the total number of coupons enriched.

**Table 2:** *Listeria monocytogenes* concentration reductions (mean ± standard deviation in log CFU/coupon) on food contact surfaces under conditions of 45–55% RH, 22 °C enumerated on non-selective media (10 samples in each cell).

Days	Polycarbonate	Polypropylene	PVC	Rubber	Stainless Steel
0	0.00 ± 0.00 aA ^a^	0.00 ± 0.00 aA	0.00 ± 0.00 aA	0.00 ± 0.00 aA	0.00 ± 0.00 aA
0.06	0.81 ± 0.34 bB	0.72 ± 0.15 bB	0.80 ± 0.35 bB	0.73 ± 0.40 bB	1.79 ± 0.43 aB
0.25	1.25 ± 0.54 bC	2.07 ± 0.90 aC	1.77 ± 0.75 abC	1.89 ± 0.94 abC	2.16 ± 0.61 aBC
1	1.63 ± 0.27 cC	2.52 ± 0.82 aCD	2.35 ± 0.83 abD	1.89 ± 0.76 bcC	2.38 ± 0.44 abCD
2	2.28 ± 0.37 bcD	2.22 ± 0.35 cCD	2.68 ± 0.55 abD	2.38 ± 0.58 bcCD	2.82 ± 0.55 aCDE
3	2.29 ± 0.39 cD	2.61 ± 0.27 bD	3.51 ± 0.42 aE	2.86 ± 0.33 bDE	2.63 ± 0.22 bDEF
7	2.29 ± 0.10 cD	3.32 ± 0.64 bE	3.70 ± 0.33 aE	3.31 ± 0.49 bEF	2.99 ± 0.28 bEF
10	2.94 ± 0.50 cE	3.38 ± 0.52 bcE	4.63 ± 0.54 aF	3.76 ± 1.06 bF	3.16 ± 0.41 cF
14	3.18 ± 0.18 cE	3.55 ± 0.51 bcE	4.69 ± 0.57 aF (1/1)	4.45 ± 0.77 aG (3/3)	3.91 ± 0.73 bG
21	4.06 ± 0.65 bF (2/3) ^b^	4.93 ± 0.40 aF (2/3)	5.06 ± 0.36 aFG (0/5)	4.76 ± 0.52 aGH (4/4)	4.94 ± 0.63 aH (1/3)
30	4.86 ± 0.42 cG (2/4)	5.29 ± 0.23 abF (0/5)	5.31 ± 0.08 abFG (1/7)	5.10 ± 0.29 bcH (1/3)	5.42 ± 0.35 aHI (2/5)
60	5.07 ± 0.42 bG (2/8)	5.12 ± 0.42 bF (4/4)	5.31 ± 0.08 bG (0/8)	5.22 ± 0.25 bH (1/6)	5.64 ± 0.08 abI (2/10)
90	5.27 ± 0.00 aG (2/10)	5.30 ± 0.19 aF (1/6)	5.34 ± 0.00 aG (2/10)	5.31 ± 0.08 aH (2/9)	5.66 ± 0.00 aI (1/10)

^a^ Lowercase letters indicate significant differences (*p* ≤ 0.05) at a single time point (rows). Capital letters indicate a significant difference (*p* ≤ 0.05) within material type over time (columns). ^b^ Parentheses express the number of coupons with a positive enrichment result over the total number of coupons enriched.

**Table 3:** Results of a log-linear model for daily die-off rates (log CFU/coupon/day) of *Salmonella* and *Listeria monocytogenes* onto food contact surfaces.

Surface by Pathogen		Effect Estimate ^a^	95% Confidence Interval	*p*-Value
*Salmonella* (reference is Rubber)				
	Polycarbonate	−0.012	−0.017, −0.007	<0.001
	Polypropylene	−0.012	−0.016, −0.007	<0.001
	PVC	−0.009	−0.014, −0.005	<0.001
	Stainless Steel	−0.009	−0.013, −0.004	0.001
*Listeria monocytogenes* (reference is Rubber)				
	Polycarbonate	−0.004	−0.012, 0.004	0.34
	Polypropylene	0.000	−0.007, 0.008	0.92
	PVC	0.003	−0.005, 0.011	0.46
	Stainless Steel	−0.002	−0.010, 0.006	0.57

^a^ Effect estimates can be interpreted as the difference in daily die-off rate due to a change from the reference level to a different level.

**Table 4:** Model parameters for the log-linear and biphasic regression models used to describe the die-off of *Salmonella* and *Listeria monocytogenes* on food contact surfaces.

Surface by Pathogen		Linear		Biphasic			
		Intercept	k_max_ ^a^	Intercept	f ^b^	k_max1_ ^c^	k_max2_ ^d^
*Salmonella*							
	All	5.614	0.047	6.446	−1.379	0.828	0.037
	Polycarbonate	5.537	0.050	6.245	−1.259	0.600	0.041
	Polypropylene	5.556	0.050	6.508	−1.443	1.368	0.041
	PVC	5.478	0.048	6.535	−1.582	1.585	0.038
	Rubber	5.885	0.038	6.628	−1.319	0.608	0.028
	Stainless Steel	5.615	0.047	6.425	−1.395	0.713	0.037
*Listeria monocytogenes*							
	All	4.329	0.047	5.709	−2.723	0.748	0.023
	Polycarbonate	4.686	0.050	5.876	−2.185	0.785	0.033
	Polypropylene	4.341	0.046	5.633	−2.630	0.660	0.022
	PVC	3.917	0.043	5.748	−3.574	0.980	0.013
	Rubber	4.236	0.046	5.744	−3.067	0.757	0.019
	Stainless Steel	4.463	0.048	5.571	−2.161	0.641	0.030

^a^ Rate of decline (log CFU/coupon/day). ^b^ Population that dies off at the rate of k_max1_. ^c^ Rate of decline (log CFU/coupon/day) before the breakpoint. ^d^ Rate of decline (log CFU/coupon/day) after the breakpoint.

**Table 5:** Goodness of fit for the log-linear and biphasic regression models used to describe the die-off of *Salmonella* and *Listeria monocytogenes* on food contact surfaces.

Surface by Pathogen		Linear		Biphasic	
		AIC ^a^	dAIC ^b^	AIC	dAIC
*Salmonella*					
	All	2900.00	726.35	2173.68	0.00
	Polycarbonate	546.51	131.46	415.05	0.00
	Polypropylene	555.90	187.31	368.59	0.00
	PVC	605.97	184.19	421.78	0.00
	Rubber	521.60	168.43	353.17	0.00
	Stainless Steel	529.56	197.13	332.43	0.00
*Listeria monocytogenes*					
	All	4209.99	1170.05	3039.94	0.00
	Polycarbonate	733.72	237.36	496.36	0.00
	Polypropylene	838.81	215.74	623.07	0.00
	PVC	914.41	401.68	512.73	0.00
	Rubber	877.05	281.95	595.09	0.00
	Stainless Steel	788.28	162.82	625.46	0.00

^a^ Akaike Information Criterion. ^b^ Delta Akaike Information Criterion.

## Data Availability

The raw data of this article will be made available by the authors on request.

## Supplementary Materials

The following supporting information can be downloaded at: https://www.mdpi.com/article/10.3390/foods14183247/s1, Table S1. *Salmonella* concentration (mean ± standard deviation in log CFU/coupon) on food contact surfaces under conditions of 45–55% RH, 22 °C enumerated on selective media (n = 10 each cell); Table S2. *Listeria monocytogenes* concentration (mean ± standard deviation in log CFU/coupon) on food contact surfaces under conditions of 45–55% RH, 22 °C enumerated on selective media (n = 10 each cell); Table S3. *Salmonella* concentration (mean ± standard deviation in log CFU/coupon) on food contact surfaces under conditions of 45–55% RH, 22 °C enumerated on non-selective media (n = 10 each cell); Table S4. *Listeria monocytogenes* concentration (mean ± standard deviation in log CFU/coupon) on food contact surfaces under conditions of 45–55% RH, 22 °C enumerated on non-selective media (n = 10 each cell).
