# Supplementary material for: Survival of Salmonella and Listeria monocytogenes on Food Contact Surfaces in Produce Packinghouses
Source: Foods. 2025 Sep 18;14(18):3247. doi: 10.3390/foods14183247 (PMC12469379; doi:10.3390/foods14183247)
Supplement: Supplementary file 1 [file foods-14-03247-s001.zip › foods-3842627-supplementary.pdf]

Supplemental Materials

Table S1. *Salmonella* concentration (mean ± standard deviation in log CFU/coupon) on food contact surfaces under conditions of 45 - 55% RH, 22°C enumerated on selective media (n=10 each cell)

Days	Polycarbonate	Polypropylene	PVC	Rubber	Stainless steel
0	6.60 ± 0.29 bA[a]	6.63 ± 0.21 bA	6.50 ± 0.36 aA	6.84 ± 0.09 aA	6.57 ± 0.26 abA
0.06	5.63 ± 0.31 cB	5.83 ± 0.49 aB	5.81 ± 0.18 bB	6.00 ± 0.20 bB	5.84 ± 0.13 bB
0.25	5.22 ± 0.49 abB	5.46 ± 0.27 bC	5.30 ± 0.53 abB	5.86 ± 0.10 aB	5.57 ± 0.21 abBC
1	5.08 ± 0.61 aB	4.97 ± 0.29 bD	4.91 ± 0.33 bC	5.55 ± 0.33 aB	5.29 ± 0.55 aBC
2	4.90 ± 0.64 aB	4.57 ± 0.20 bE	4.75 ± 0.26 bCD	5.15 ± 0.42 aBC	5.08 ± 0.41 aC
3	4.57 ± 0.51 aC	4.19± 0.24 cEF	4.32 ± 0.48 abD	4.97 ± 0.16 aCD	4.63 ± 0.24 bcD
7	4.39 ± 0.52 aCD	4.09 ± 0.57 aF	4.04 ± 0.68 aD	4.48 ± 0.49 aDE	4.65 ± 0.44 aDE
10	3.78 ± 0.74 aDE	3.86 ± 0.61 aFG	3.77 ± 0.93 aD	4.26 ± 0.37 aDE	4.06 ± 0.38 aEF
14	3.74 ± 0.81 abDE	3.44 ± 0.72 bcG	3.30 ± 1.08 abcE	4.21 ± 0.49 aEF	3.91 ± 0.13 cFG
21	3.55 ± 0.74 aE	3.00 ± 0.18 cH	2.92 ± 0.35 cF	3.95 ± 0.19 bEF	3.78 ± 0.19 bFG
30	3.10 ± 0.69 bF	2.59 ± 1.15 bI	2.71 ± 0.97 bF	3.96 ± 0.37 aF	3.48 ± 0.36 bG
60	2.21 ± 0.74 aG (1/3)[b]	2.31 ± 0.92 aI (1/4)	2.17 ± 0.84 aG (4/7)	3.13 ± 0.90 aG	2.45 ± 0.81 aH
90	<1.30 aH (1/10)	<1.30 aJ (3/10)	<1.30 aH (2/10)	1.78 ± 0.75 aH (4/5)	<1.30 aI (3/8)

[a] Lowercase letters indicate significant differences ($p \leq 0.05$) at a single time point (rows). Capital letters indicate a significant difference ($p \leq 0.05$) within material type over time (columns).

[b] Parenthesis express the number of coupons with a positive enrichment result over the total number of coupons enriched.

Table S2. *Listeria monocytogenes* concentration (mean ± standard deviation in log CFU/coupon) on food contact surfaces under conditions of 45 - 55% RH, 22°C enumerated on selective media (n=10 each cell)

Days	Polycarbonate	Polypropylene	PVC	Rubber	Stainless steel
0	5.79 ± 0.10 cA[a]	6.19 ± 0.26 abA	6.10 ± 0.18 abA	6.04 ± 0.20 bA	6.23 ± 0.15 aA
0.06	4.68 ± 0.23 bB	4.86 ± 0.34 bB	4.95 ± 0.43 abB	5.23 ± 0.20 aB	4.66 ± 0.40 bB
0.25	4.45 ± 0.53 aB	4.25 ± 0.56 aC	4.38 ± 0.60 aC	4.15 ± 0.90 aC	4.20 ± 0.67 aC
1	3.55 ± 0.37 aC	3.76 ± 0.61 aD	3.70 ± 0.43 aD	3.94 ±0.94 aCD	3.51 ± 0.69 aD
2	3.55 ± 0.43 aC	3.07 ± 0.61 aE	3.11 ± 0.36 aE	3.43 ± 1.00 aD	3.21 ± 0.41 aD
3	3.08 ± 0.37 aD	3.06 ± 0.29 aE	2.38 ± 0.49 bF	2.49 ± 0.57 bE	2.65 ± 0.46 bE
7	2.60 ± 0.51 abE	2.30 ±0.62 abcF	2.08 ± 0.56 cF	2.65 ± 0.28 aE	2.16 ± 0.50 bcF
10	1.85 ± 0.36 aF	1.80 ± 0.54 aG	1.43 ± 0.26 bG	1.51 ± 0.34 abF	1.77 ± 0.49 abF
14	1.35 ± 0.18 aG (6/6)[b]	1.36 ± 0.24 aH (5/7)	<1.30 aG (4/9)	1.30 ± 0.10 aF (4/5)	1.28 ± 0.08 aG (4/6)
21	<1.30 aG (3/9)	1.30 ± 0.18 aH (0/9)	<1.30 aG (2/9)	<1.30 aF (3/6)	1.28 ± 0.08 aG (2/7)
30	<1.30 aG (2/7)	<1.30 aH (0/8)	<1.30 aG (1/9)	<1.30 aF (2/10)	<1.30 aG (7/10)
60	<1.30 aG (2/10)	<1.30 aH (1/8)	<1.30 aG (0/10)	<1.30 aF (0/10)	<1.30 aG (2/10)
90	<1.30 aG (0/10)	<1.30 aH (1/10)	<1.30 aG (0/10)	<1.30 aF (4/10)	<1.30 aG (0/10)

[a] Lowercase letters indicate significant differences ($p \leq 0.05$) at a single time point (rows). Capital letters indicate a significant difference ($p \leq 0.05$) within material type over time (columns).
[b] Parenthesis express the number of coupons with a positive enrichment result over the total number of coupons enriched.

Table S3. *Salmonella* concentration (mean ± standard deviation in log CFU/coupon) on food contact surfaces under conditions of 45 - 55% RH, 22°C enumerated on non-selective media (n=10 each cell)

Days	Polycarbonate	Polypropylene	PVC	Rubber	Stainless steel
0	6.82 ± 0.09 cA[a]	6.92 ± 0.12 abA	6.95 ± 0.10 aA	6.97 ± 0.13 aA	6.84 ± 0.07 bcA
0.06	5.94 ± 0.13 cB	6.25 ± 0.28 abB	6.19 ± 0.15 bB	6.44 ± 0.34 aB	6.39 ± 0.24 abB
0.25	5.83 ± 0.22 bBC	5.86 ± 0.20 bBC	5.92 ± 0.18 bB	6.28 ± 0.16 aB	5.82 ± 0.19 bC
1	5.57 ± 0.38 bcBCD	5.50 ± 0.26 bcCD	5.33 ± 0.38 cC	6.00 ± 0.21 aBC	5.70 ± 0.35 bCD
2	5.35 ± 0.44 bCD	5.15 ± 0.22 bDE	5.18 ± 0.45 bC	5.49 ± 0.27 aC	5.38 ± 0.23 bDE
3	5.11 ± 0.55 abDE	4.91 ± 0.22 bE	4.89 ± 0.39 bCD	5.28 ± 0.21 aD	5.09 ± 0.19 abEF
7	4.81 ± 0.54 abEF	4.84 ± 0.57 abE	4.48 ± 0.52 bDE	5.10 ± 0.54 aDE	4.6 ± 0.31 abFG
10	4.42 ± 0.57 bFG	4.67 ± 0.39 abE	4.50 ± 0.58 bDE	5.96 ± 0.52 aDEF	4.45 ± 0.41 bGH
14	4.25 ± 0.37 bG	4.19 ± 0.47 bF	4.25 ± 0.49 bE	4.84 ± 0.39 bDEF	4.33 ± 0.51 aHI
21	4.26 ± 0.51 bGH	4.13 ± 0.47 bF	4.13 ± 0.57 bEF	4.67 ± 0.29 aEF	4.28 ± 0.41 abHI
30	3.73 ± 0.57 bH	3.90 ± 0.47 bF	3.62 ± 0.60 bFG	4.60 ± 0.19 aF	3.98 ± 0.25 bI
60	2.65 ± 1.05 bI (3/3)[b]	2.71 ± 1.13 bG (4/4)	3.12 ± 1.00 abG (4/4)	3.88 ± 0.42 aG	3.17 ± 0.79 abJ
90	<1.30 bJ (1/10)	<1.30 bH (2/10)	<1.30 bH (2/10)	2.59 ± 1.11 aH (2/3)	1.50 ± 0.64 bK (5/9)

[a] Lowercase letters indicate significant differences ($p \leq 0.05$) at a single time point (rows). Capital letters indicate a significant difference ($p \leq 0.05$) within material type over time (columns).

[b] Parenthesis express the number of coupons with a positive enrichment result over the total number of coupons enriched.

Table S4. *Listeria monocytogenes* concentration (mean ± standard deviation in log CFU/coupon) on food contact surfaces under conditions of 45 - 55% RH, 22°C enumerated on non-selective media (n=10 each cell)

Days	Polycarbonate	Polypropylene	PVC	Rubber	Stainless steel
0	6.53 ± 0.29 bA[a]	6.65 ± 0.21 bA	6.59 ± 0.25 bA	6.59 ± 0.26 bA	6.92 ± 0.11 aA
0.06	5.72 ± 0.34 aB	5.93 ± 0.15 aB	5.79 ± 0.35 aB	5.86 ± 0.40 aB	5.12 ± 0.43 bB
0.25	5.28 ± 0.54 aC	4.58 ± 0.90 bC	4.82 ± 0.75 abC	4.70 ± 0.94 abC	4.75 ± 0.61 abBC
1	4.90 ± 0.27 aC	4.13 ± 0.82 bCD	4.24 ± 0.83 bD	4.69 ± 0.75 abC	4.54 ± 0.44 abCD
2	4.25 ± 0.37 abD	4.43 ± 0.35 aCD	3.91 ± 0.55 bD	4.21 ± 0.58 abCD	4.10 ± 0.55 abDE
3	4.24 ± 0.39 aD	4.05 ± 0.27 aD	3.08 ± 0.43 cE	3.73 ± 0.33 bDE	4.29 ± 0.22 aDEF
7	4.24 ± 0.10 aD	3.33 ± 0.64 bE	2.89 ± 0.33 cE	3.28 ± 0.49 bEF	3.93 ± 0.28 aEF
10	3.59 ± 0.50 aE	3.27 ± 0.52 abE	1.96 ± 0.54 cF	2.83 ± 1.06 bF	3.76 ± 0.41 aF
14	3.35 ± 0.18 aE	3.10 ± 0.51 aE	1.90 ± 0.56 bF (1/1)	2.14 ± 0.77 bG (3/3)	3.01 ± 0.73 aG
21	2.47 ± 0.65 aF (2/3)[b]	1.72 ± 0.40 bF (2/3)	1.53 ± 0.36 bFG (0/5)	1.83 ± 0.52 bGH (4/4)	1.98 ± 0.63 bH (1/3)
30	1.67 ± 0.42 aG (2/4)	1.37 ± 0.23 bF (0/5)	1.28 ± 0.08 bG (1/7)	1.49 ± 0.29 abH (1/3)	1.50 ± 0.35 abI (2/5)
60	1.46 ± 0.42 abGH (2/8)	1.54 ± 0.42 aF (4/4)	1.28 ± 0.08 abG (0/8)	1.37 ± 0.35 abH (1/6)	1.28 ± 0.08 bI (2/10)
90	<1.30 bH (2/10)	1.36 ± 0.19 aF (1/6)	<1.30 bG (2/10)	1.31 ± 0.08 bH (2/9)	<1.30 bI (1/10)

[a] Lowercase letters indicate significant differences ($p \leq 0.05$) at a single time point (rows). Capital letters indicate a significant difference ($p \leq 0.05$) within material type over time (columns).

[b] Parenthesis express the number of coupons with a positive enrichment result over the total number of coupons enriched.
